# Prevalence and correlates of suicidal ideation among older adults attending primary care clinics in Wuhan, China: A multicenter cross-sectional study

**DOI:** 10.3389/fpsyt.2022.1003810

**Published:** 2022-09-09

**Authors:** Xiao-Min Zhu, Yan-Min Xu, Zong-Qin Wang, Bao-Liang Zhong

**Affiliations:** ^1^Department of Psychiatry, Suzhou Guangji Hospital, The Affiliated Guangji Hospital of Soochow University, Suzhou, China; ^2^Department of Psychiatry, Wuhan Mental Health Center, Wuhan, China; ^3^Department of Clinical Psychology, Wuhan Hospital for Psychotherapy, Wuhan, China

**Keywords:** older adults, primary care, suicidal ideation, cross-sectional survey, China

## Abstract

**Background:**

Primary care represents an ideal setting for screening for and managing suicidal older adults but the clinical epidemiology of suicidal ideation in Chinese older primary care patients remains unclear. This study investigated the prevalence and correlates of suicidal ideation in older Chinese adults receiving primary care.

**Methods:**

This multicenter cross-sectional survey included a total of 769 older adults (≥65 years) from seven urban and six rural primary care clinics in Wuhan, China. The presence of depressive symptoms and suicidal ideation was assessed with the Geriatric Depression Scale and a single-item question “In the past 12 months, did you think about ending your life?,” respectively.

**Results:**

The 12-month prevalence of suicidal ideation in older primary care patients was 16.6%. Significant correlates of suicidal ideation were poor economic status (vs. good, OR = 2.80, *P* = 0.008), heart disease (OR = 2.48, *P* = 0.005), chronic gastric ulcer (OR = 3.55, *P* = 0.012), arthritis (OR = 2.10, *P* = 0.042), and depressive symptoms (OR = 11.29, *P* < 0.001).

**Conclusions:**

Suicidal ideation is common among older adults attending Chinese primary care clinics. It is necessary to integrate psychological crisis intervention into primary care to prevent late-life suicide.

## Introduction

Despite the declining time-trend in national elderly suicide rates in recent decades, suicide in older adults remains a significant public health concern in China because of its higher elderly suicide rate compared to most major East Asian and Western countries/regions, largest number of older adults in the world, and rapid increase of aging population (1–6). Given the complexity of late-life suicide, a multi sectoral suicide prevention strategy is needed for effective suicide prevention in older adults (7–9).

In Western countries, Hong Kong SAR, and mainland China, as high as 60–80% of the older suicide completers visited their primary care physicians (PCPs) in the preceding month before committing suicide (10–13). Given the wide availability of primary care services and the frequent contacts between PCPs and older adults, primary care represents an ideal setting for screening for and managing suicidal older adults (14–16). Accordingly, one essential component of the national strategies for suicide prevention of most countries in the world, as recommended by the World Health Organization, is to provide and maintain gatekeeper training programs to PCPs to improve their capacity to identify persons who are at-risk of suicide and refer them to mental health specialists when necessary (7).

To facilitate the timely detection, suicide risk assessment, and early initiation of the intervention of suicidal older adults in primary care settings, it is necessary to understand the clinical epidemiology of suicidal behaviors in older primary care patients. Until now, suicidal behaviors, in particular suicidal ideation, have been extensively studied in older primary care adults in Western countries (17–23). These studies assessed the presence of suicidal ideation with various instruments (i.e., suicidality subscale of the Depressive Symptom Inventory and suicidal item of the nine-item Patient Health Questionnaire) and reported a wide range of prevalence estimates (0.7–23.3%) (18, 21, 23). A variety of factors were found to be significantly associated with suicidal ideation in this population, including male sex, living alone, financial strain, family history of suicide, depressive symptoms, pain, and poor physical health (17, 18, 23).

Findings from psychological autopsy studies in both Western countries and China have reported the greater risk of suicide in older adults with physical illnesses (24, 25); for example, congestive heart failure, chronic obstructive lung disease (COPD), seizure disorder, and urinary incontinence are associated with 1.7-, 1.6-, 3.0-, and 2.0-fold increases in odds of suicide in older adults, respectively. However, one significant knowledge gap of the above studies is that few data are available regarding the risk of suicidal behaviors in older primary care patients according to physical conditions, which may help the identification of high-risk subgroups in this physically ill population. In addition, accumulating empirical evidence has shown the important role of social disconnectedness-related factors in the etiology of later-life suicide, which are potentially modifiable and include lack of close friends, living alone, feelings of loneliness, no trusted friends, insufficient number family relatives, and inadequate social support (12, 26–30). Nevertheless, few prior studies have examined the contributions of these factors to suicidal behaviors in older primary care patients.

Although suicidal ideation is less prevalent in old age-group than younger age-groups, older adults, once they have suicidal thoughts, are more likely to commit suicide with greater suicidal intention and by using more immediately lethal means (1, 31–34). These data suggest that screening for suicidal ideation is the first essential step toward effective prevention of late-life suicide. However, to the best of our knowledge, few studies have investigated the clinical epidemiology of suicidal ideation in older adults undergoing primary care services in China. This study filled the above-mentioned knowledge gaps by investigating the prevalence of suicidal ideation and its associated factors in older primary care patients in Wuhan, the largest city in Hubei province and the most populous city in Central China (35). Factors to be examined included major medical conditions and some social disconnectedness-related factors.

## Methods

### Sampling and subjects

Subjects were 791 older adults who were consecutively recruited from 13 district-center public primary care clinics (seven urban and six rural) in Wuhan, 65 years old or over, receiving outpatient treatment at these selected clinics during the survey period from October 2015 to November 2016, and voluntary to participate in this study. Details of the sampling and subject inclusion have been described elsewhere (14, 36–38). Wuhan has 13 districts: seven urban and six rural. Considering the geographic representativeness of the study sample, we purposively selected one primary care clinic from each district, which was located in or nearest to the center of the most populous area of the district.

Prior to the formal study, the survey protocol was approved by the Institutional Review Board of Wuhan Mental Health Center (approval number: WMHC-IRB-S065). All subjects and their guardians (if necessary) provided informed consent before the interview.

### Procedures and instruments

Trained PCPs collected the questionnaire data *via* face-to-face interviews with the older primary care patients. Demographic factors in the questionnaire included sex, age, educational attainment, marital status, self-rated economic status (good, fair, poor), main occupation engaged in before the older adulthood (physical vs. mental), and current residence place (urban vs. rural).

Lifestyle factors included currently smoking and the habit of regular physical activity (36). We used a checklist to assess the presence of 11 major medical conditions and two sensory impairments: hypertension, diabetes, heart diseases, stroke and other cerebrovascular diseases, COPD, tuberculosis, chronic gastric ulcer, Parkinson's disease, anemia, hepatitis cirrhosis, arthritis, hearing impairment, and vision impairment. Hearing impairment was present if the interviewer must speak more loudly than normal to let the patients know what the interviewer was saying, while vision impairment was present if the patient endorsed having difficulties in watching movies or TV shows (32, 36).

Social disconnectedness-related factors included number of living adult children, the status of living alone, feelings of loneliness, self-rated relationship with family members (good, fair, poor), and self-rated relationship with non-family associates (good, fair, poor). A single-item question was used to assess feelings of loneliness: “How often do you feel lonely?” with five response options: always, often, sometimes, seldom, and never. Respondents who reported felt lonely at least sometimes were those having feelings of loneliness (38).

Depressive symptoms were evaluated with validated Chinese version of the Geriatric Depression Scale with a total score of five or greater denoting clinically significant depressive symptoms (37, 39). One-year suicidal ideation was assessed with a single question “In the past 12 months, did you think about ending your life?”(40).

### Statistical analysis

Prevalence of suicidal ideation was calculated. Chi-square test was used to compare rates of suicidal ideation between/across subgroups according to demographic and other characteristics. Unconditional binary logistic regression was used to identify correlates of suicidal ideation, which included all significant factors from the Chi-square test as the independent variables and employed backward elimination to select the final set of correlates. Associations between correlates and suicidal ideation were measured by using Odds Ratios (ORs) and their 95% confidence intervals (95% CIs). SPSS software, version 16.0, was used for analyzing the data. A two-sided *P* < 0.05 was defined as statistically significant.

## Results

In total, 791 older primary care adults were invited to join the study and 769 completed the survey questionnaire. Reasons for non-completion of the survey included: refusal to join in the survey (*n* = 10), very severe cognitive impairment (*n* = 5), the withdrawal of informed consent (*n* = 6), and missing data on variables of interest (*n* = 1). The average age of the study sample was 72.9 years (standard deviation = 6.1, range = 65–97) and 414 (53.8%) were women. Table 1 displays the characteristics of the survey sample and prevalence rates of suicidal ideation by sample characteristics.

**Table 1:** Characteristics of the sample of older primary care patients and prevalence rates of suicidal ideation by sample characteristics.

	**Characteristics**	**No. of older adults**	**No. of older adults with suicidal ideation**	**Prevalence (%)**	**χ^2^**	***P*-value**
Sex	Male	355	56	15.8	0.360	0.548
	Female	414	72	17.4		
Age (years)	65–74	493	85	17.2	0.352	0.553
	75+	276	43	15.6		
Education	Illiterate	181	43	23.8	10.944	0.004
	Primary school	217	38	17.5		
	Middle school and above	371	47	12.7		
Marital status	Married	534	87	16.3	0.157	0.692
	Others^*^	235	41	17.4		
Self-rated financial status	Good	137	18	13.1	33.770	<0.001
	Fair	543	76	14.0		
	Poor	89	34	38.2		
Main occupation before older adulthood	Mental labor	223	26	11.7	5.627	0.018
	Manual labor	546	102	18.7		
Residence place	Urban	414	63	15.2	1.317	0.251
	Rural	355	65	18.3		
Current smoking	No	647	109	16.8	0.120	0.729
	Yes	122	19	15.6		
Regular physical activity	No	438	66	15.1	1.823	0.177
	Yes	331	62	18.7		
Hypertension	No	397	65	16.4	0.044	0.834
	Yes	372	63	16.9		
Diabetes	No	653	104	15.9	1.611	0.204
	Yes	116	24	20.7		
Heart disease	No	688	105	15.3	9.009	0.003
	Yes	81	23	28.4		
Stroke and other cerebrovascular diseases	No	705	110	15.6	6.631	0.010
	Yes	64	18	28.1		
Chronic obstructive pulmonary disease	No	724	120	16.6	0.044	0.833
	Yes	45	8	17.8		
Cancer	No	764	127	16.6	0.041	0.840
	Yes	5	1	20.0		
Tuberculosis	No	767	127	16.6	1.608	0.205
	Yes	2	1	50.0		
Chronic gastric ulcer	No	744	119	16.0	6.977	0.008
	Yes	25	9	36.0		
Parkinson's disease	No	764	126	16.5	1.979	0.160
	Yes	5	2	40.0		
Anemia	No	761	126	16.6	0.407	0.524
	Yes	8	2	25.0		
Hepatitis cirrhosis	No	767	127	16.6	1.608	0.205
	Yes	2	1	50.0		
Arthritis	No	709	110	15.5	8.366	0.004
	Yes	60	18	30.0		
Hearing impairment	No	739	125	16.9	0.994	0.319
	Yes	30	3	10.0		
Vision impairment	No	692	112	16.2	1.054	0.305
	Yes	77	16	20.8		
Self-rated family relationship	Good	605	86	14.2	12.075	0.001
	Fair and poor^**^	164	42	25.6		
Self-rated non-family relationship	Good	535	82	15.3	2.201	0.138
	Fair and poor^**^	234	46	19.7		
Feelings of loneliness	No	566	67	11.8	35.717	<0.001
	Yes	203	61	30.0		
Living alone	No	686	116	16.9	0.321	0.571
	Yes	83	12	14.5		
Number of living adult children	0	20	6	30.0	3.807	0.283
	1	87	17	19.5		
	2	233	34	14.6		
	3+	429	71	16.6		
Depressive symptoms	No	531	30	5.6	149.5	<0.001
	Yes	238	98	41.2		

* “Others” included never married, separated, divorced, widowed, cohabitating, and remarried.

** Because of the very small numbers of the category of “poor” relationship (n <10), “poor” and “fair” were merged into one category.

Altogether, 128 older adults endorsed suicidal ideation during the past year and the corresponding 1-year prevalence of suicidal ideation was 16.6%.

Results from Chi-square test (Table 1) show that significantly higher rates of suicidal ideation were observed in illiterate respondents (vs. middle school and above), in respondents with poor economic status (vs. good), in respondents who engaged in physical labor (vs. mental labor) before the older adulthood, in respondents suffering heart disease (vs. no), in respondents suffering stroke and other cerebrovascular diseases (vs. no), in respondents suffering chronic gastric ulcer (vs. no), in respondents suffering arthritis (vs. no), in respondents with fair and poor family relationship (vs. good), in respondents having feelings of loneliness (vs. no), and in respondents having depressive symptoms (vs. no).

Multiple logistic regression analysis identified five factors significantly associated with suicidal ideation in older primary care patients (Table 2): poor economic status (vs. good, OR = 2.80, *P* = 0.008), heart disease (OR = 2.48, *P* = 0.005), chronic gastric ulcer (OR = 3.55, *P* = 0.012), arthritis (OR = 2.10, *P* = 0.042), and depressive symptoms (OR = 11.29, *P* < 0.001).

**Table 2:** Correlates of suicidal ideation in older primary care patients.

**Factor**	**Risk level**	**Reference level**	**Co-efficient**	**Standard error**	**χ^2^**	***P*-value**	**OR (95% CI)**
Self-rated financial status	Poor	Good	1.028	0.389	6.982	0.008	2.80 (1.30, 5.99)
Heart disease	Yes	No	0.909	0.320	8.061	0.005	2.48 (1.33, 4.65)
Chronic gastric ulcer	Yes	No	1.265	0.505	6.275	0.012	3.55 (1.32, 9.54)
Arthritis	Yes	No	0.740	0.364	4.145	0.042	2.10 (1.03, 4.28)
Depressive symptoms	Yes	No	2.424	0.240	102.159	<0.001	11.29 (7.06, 18.07)

## Discussion

Given the limited mental health service and crisis intervention resources in China, empirical data on the clinical epidemiology of suicidal ideation in older primary care patients would inform the development of late-life suicide prevention programs in Chinese primary care settings. To the best of our knowledge, this is the first study in China that examined the prevalence and correlates of suicidal ideation among older Chinese adults receiving primary care. The main findings of this study are the 16.6% prevalence of 1-year suicidal ideation and its significant associations with poor economic status, heart disease, chronic gastric ulcer, arthritis, and depressive symptoms.

Compared to the 2.6 and 5.7% prevalence of 1-year suicidal ideation in urban and rural community-residing older Chinese adults, respectively (41, 42), our study found a much higher prevalence of suicidal ideation in older primary care adults in China. The high risk of suicidal ideation in older Chinese adults attending primary care clinics should be primarily due to their prevailing physical health problems, because poor physical health has been associated with poor mental health, i.e., acute stress caused by the fracture and post-stroke depression, which in turn results in the elevated risk of suicidal behaviors (1, 14, 24, 25). Further, there is evidence that the treatment rate of major depression in older adults receiving primary care in China is extremely low, <1% (43). Therefore, the much higher prevalence of suicidal ideation might be the result of untreated late-life depression in primary care settings in China.

In line with earlier studies (17, 18, 23, 40, 44), we confirmed significant associations between poor financial status, depressive symptoms, and suicidal ideation in Chinese older primary care patients. Nevertheless, this study did not find significant associations of suicidal ideation with disconnectedness-related factors in the final step of multiple logistic regression model despite significant associations between fair and poor family relationship and feelings of loneliness and suicidal ideation in the Chi-square test. We speculate that this does not indicate that disconnectedness-related factors did not contribute to suicidal ideation; rather, the effects of these factors may be relative weak and be masked by the prevailing physical health problems of this population.

The elevated risk of suicidal ideation in individuals with heart disease, peptic ulcers, and arthritis in the general population has been previously reported (45–47). Because depression can be viewed as an antecedent for suicidal ideation and these chronic diseases have been associated with depression (45, 48, 49), our findings on the three major medical conditions as significant correlates of suicidal ideation are expected. Nevertheless, it seems that depression is not the only bridge that links major medical conditions and suicidal ideation together because both depressive symptoms and major medical conditions were significant and independent correlates in the final regression model. We speculated that major medical conditions might increase the risk of suicidal ideation *via* other pathways such as pain and psychological distress.

The main limitation of this study is the methodology of cross-sectional survey, which cannot ascertain the causality between identified correlates and suicidal ideation. The second limitation is no assessment of the mental health-help seeking behaviors of suicidal older adults, which is essential for the planning of crisis intervention in primary care settings in China. Third, some modifiable factors that are potentially associated with suicidal ideation in older adults such as pain and social support were not measured in this study.

In summary, in Chinese primary care settings, suicidal ideation is common among older patients. Given the potentially high risk of suicide in suicidal older adults, it is necessary to integrate psychological crisis intervention into primary care to prevent late-life suicide. Services in primary care settings in China should include routinely screening for older adults at-risk of suicide, suicide risk assessment, psychosocial support, antidepressant treatment, and referral to mental health specialists when necessary. In addition, the elevated risk of suicidal ideation in older adults with several major medical conditions suggests that effective management of major medical conditions should be considered as a component of the late-life suicide prevention strategy in primary care settings.

## Data availability statement

The original contributions presented in the study are included in the article/supplementary material, further inquiries can be directed to the corresponding author.

## Ethics statement

The studies involving human participants were reviewed and approved by the Institutional Review Board of Wuhan Mental Health Center (approval number WMHC-IRB-S065). The patients/participants provided their written informed consent to participate in this study.

## Author contributions

X-MZ acquisition and analysis of data for the study, drafting the paper, and interpretation of data for the study. Y-MX and Z-QW design and acquisition of data for the study. X-MZ and B-LZ drafting the paper, revising the paper for important intellectual content, and interpretation of data for the study. All authors contributed to the article and approved the submitted version.

## Funding

This work was supported by National Natural Science Foundation of China (grant number: 71774060), 2015 Irma and Paul Milstein Program for Senior Health Awards from the Milstein Medical Asian American Partnership Foundation, Wuhan Health and Family Planning Commission (grant numbers: WX17Q30, WG16A02, and WG14C24), and Suzhou Medical Talents Program from Suzhou Health Commission (grant number: GSWS2021052).

## Conflict of interest

The authors declare that the research was conducted in the absence of any commercial or financial relationships that could be construed as a potential conflict of interest.

## Publisher's note

All claims expressed in this article are solely those of the authors and do not necessarily represent those of their affiliated organizations, or those of the publisher, the editors and the reviewers. Any product that may be evaluated in this article, or claim that may be made by its manufacturer, is not guaranteed or endorsed by the publisher.
